# Muscle *Bmal1* is Dispensable for the Progress of Neurogenic Muscle Atrophy in Mice

**DOI:** 10.5334/jcr.141

**Published:** 2016-08-19

**Authors:** Reiko Nakao, Shigeki Shimba, Katsutaka Oishi

**Affiliations:** Biological Clock Research Group, Biomedical Research Institute, National Institute of Advanced Industrial Science and Technology (AIST), Tsukuba, Ibaraki, Japan; Department of Health Science, School of Pharmacy, Nihon University, Funabashi, Chiba, Japan; Department of Applied Biological Science, Graduate School of Science and Technology, Tokyo University of Science, Noda, Chiba, Japan; Department of Computational and Medical Sciences, Graduate School of Frontier Sciences, The University of Tokyo, Kashiwa, Chiba, Japan

**Keywords:** peripheral clock, sciatic denervation, sarcopenia, gastrocnemius muscle, E3 ubiquitin ligase

## Abstract

Global deletion of aryl hydrocarbon receptor nuclear translocator-like (*Arntl*; also known as *Bmal1*), a molecular component of the circadian clock, resulted in an extreme loss of muscle mass. However, the functional role of muscle BMAL1 has not been elucidated. Here, we used muscle-specific Bmal1 knockout mice to determine whether disrupting the muscle clock exacerbates muscle atrophy induced by sciatic denervation or aging. The muscle mass of wild-type and muscle-specific *Bmal1* knockout mice decreased to a similar extent at seven days after denervation, although *Bmal1* ablation partly attenuated the upregulation of genes encoding muscle atrophy-related ubiquitin ligases, muscle atrophy F-box (MAFbx) and muscle RING finger 1 (MuRF1). A comparison of adult and elderly mice aged 7 – 8 and 23 – 24 months, respectively, confirmed that ablating muscle *Bmal1* scarcely affected the extent to which aging induced the loss of muscle mass. Muscle Bmal1 minimally affected the progression of muscle atrophy caused by sciatic denervation or aging.

## Introduction

The maintenance of muscle mass depends on the balance between protein synthesis and degradation. Aging and various pathological conditions, including denervation, unloading, diabetes, cancer and sepsis, cause progressive muscle atrophy via excessive protein degradation mainly through induction of the ubiquitin-proteasome system [1]. The muscle-specific ubiquitin ligases, muscle atrophy F-box (MAFbx) and muscle RING finger 1 (MuRF1), are upregulated during muscle atrophy and in turn produce atrophy [2,3]. Several transcription factors, including forkhead box O (FOXOs) [4,5], nuclear factor of kappa light polypeptide gene enhancer in B cells 1, p105 (NFkB) [6], and myogenin [7], transcriptionally regulate the gene expression of these ligases during muscle atrophy.

Molecular circadian clock components are evident in most peripheral tissues such as the heart, liver and skeletal muscles, whereas the master clock is located in the hypothalamic suprachiasmatic nucleus (SCN) in mammals [8]. The global knockout (KO) of aryl hydrocarbon receptor nuclear translocator-like (*Arntl*; also known as *Bmal1*), a core component of the circadian clock that encodes a basic-helix-loop-helix (bHLH)-PER-ARNT-SIM (PAS) transcription factor, results in a shortened lifespan, loss of muscle mass, reduced muscle force, mitochondrial dysfunction and disrupted myofilament architecture in mice [9,10]. Schroder et al. found that specific tension was reduced and muscle fibrosis was increased in inducible muscle-specific *Bmal1* KO (M-KO) mice at the age of 70 weeks [11]. These results imply that clock components are involved in retaining skeletal muscle mass and function. We then postulated that the extent of muscle atrophy is enhanced in M-KO mouse models of atrophy. Therefore, the present study aimed to quantify the effects of a muscle-specific deletion of BMAL1 on denervation-induced and aging-induced muscle atrophy in mice.

## Materials and Methods

### Animal care and surgical procedures

Muscle-specific *Bmal1* knockout (M-KO) mice and conditional *Bmal1* flox/flox (f/f) mice (control littermates) were generated as described [12]. In brief, a mouse line with a floxed *Bmal1* gene was crossed with mice carrying a Cre recombinase transgene under control of the muscle creatine kinase (Mck) promoter. The mice were housed with access to a standard diet (CE2: CLEA Japan Inc., Tokyo, Japan) and water *ad libitum* under a 12 h light-12 h dark cycle (LD12:12); lights on at Zeitgeber Time (ZT) 0 and lights off at ZT12. Male mice aged 7 – 8 months (adult) were sacrificed at ZT1 and ZT13 (f/f, n = 6; M-KO, n = 4 at each ZT) to evaluate the effects of a *Bmal1* deletion on circadian rhythms in the gastrocnemius (Ga) and soleus (Sol) muscles. The sciatic nerve was dissected from the left hind legs of adult male mice under anesthesia (f/f, n = 11; M-KO, n = 9) to evaluate the effects of deleting muscle *Bmal1* on denervation-induced atrophy of the Ga and Sol muscles. The right hind legs remained innervated and served as a control. The mice were sacrificed at seven days after denervation. Adult f/f (n = 10) and M-KO (n = 8) and 23 – 24-month-old (aged) f/f (n = 6) and M-KO (n = 6) mice were sacrificed to evaluate the effects of aging on atrophy of the Ga and Sol muscles. The Animal Care and Use Committees at the National Institute of Advanced Industrial Science and Technology (AIST) approved all experimental protocols described herein (Permission #2015–166).

### Real-time reverse transcription-polymerase chain reaction (RT-PCR)

**Figure 1:**
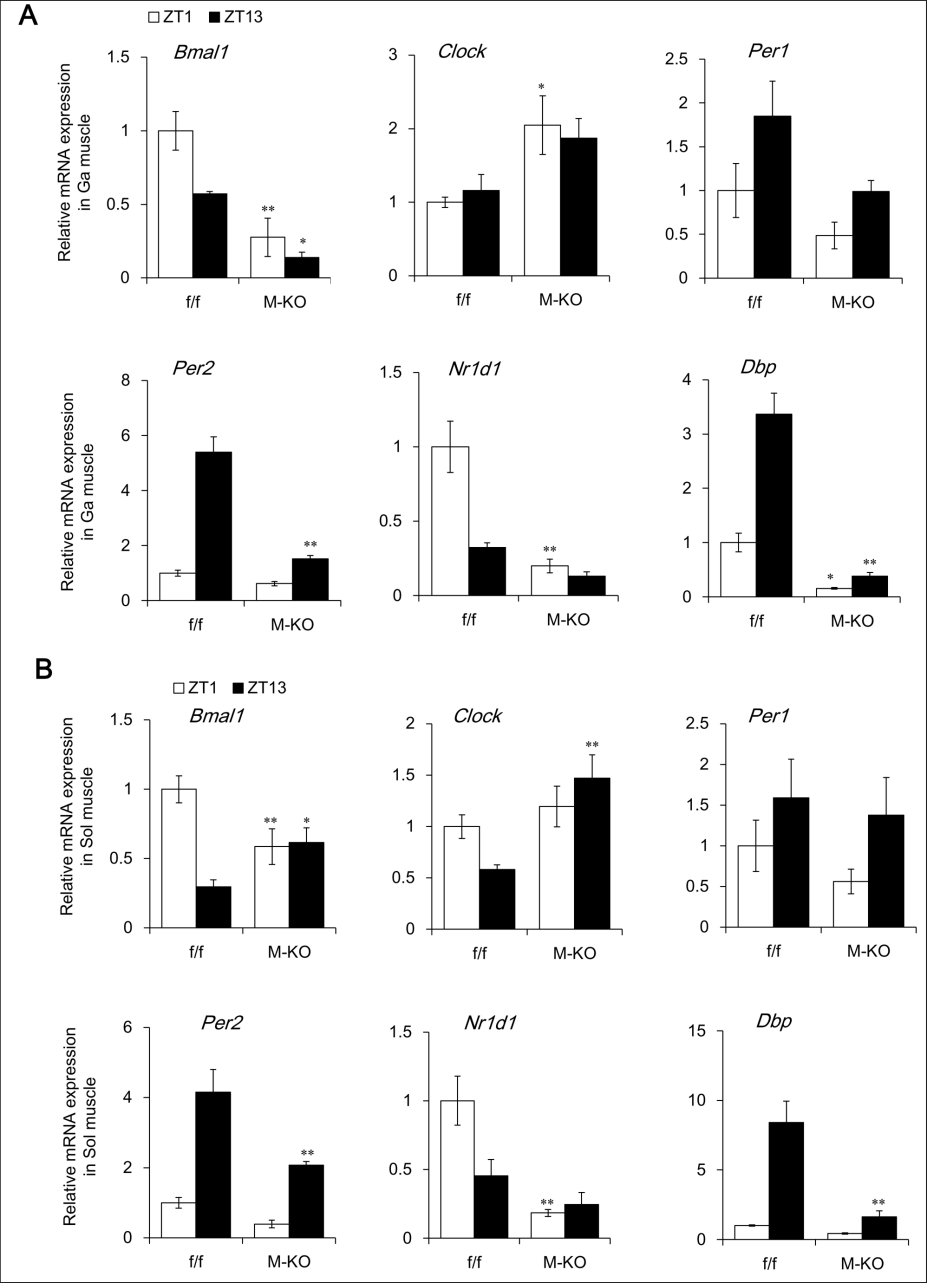
Day/night expression of clock genes. Day/night expression profiles of clock genes in gastrocnemius **(A)** and soleus **(B)** muscles of 7 – 8 month-old (adult) f/f and M-KO mice at ZT1 and ZT13. Data are shown as means ± SEM (f/f; n = 6, M-KO; n = 4 at each ZT). Value for f/f mice at ZT1 is expressed as 1.0. *P < 0.05 and **P < 0.01 for f/f vs. M-KO mice. Ga, gastrocnemius; Sol, soleus.

**Figure 2:**
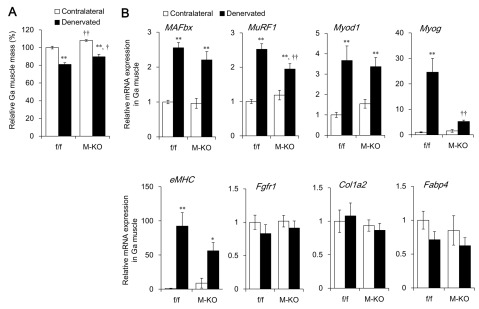
Extent of denervation-induced gastrocnemius (Ga) muscle atrophy in muscle specific-*Bmal1* knockout (M-KO) and littermate control (f/f) mice. **(A)** Relative Ga muscle mass of 7 – 8-month-old (adult) f/f and M-KO mice at seven days after denervation. Sciatic nerves in left hind legs were dissected and contralateral hind legs served as controls. Value for contralateral Ga muscle weight/body weight in f/f mice is shown as 100%. **(B)** Expression of mRNA for genes associated with muscle atrophy or degeneration in Ga muscle at seven days after denervation. Data are expressed as means ± SEM (f/f; n = 11, M-KO; n = 9). The value for contralateral muscle of f/f mice is expressed as 1.0. *P < 0.05 and **P < 0.01 for contralateral vs. denervated muscles. ^†^*P* < 0.05 and ^††^*P* < 0.01 for f/f vs. M-KO mice. Ga, gastrocnemius muscle.

**Figure 3:**
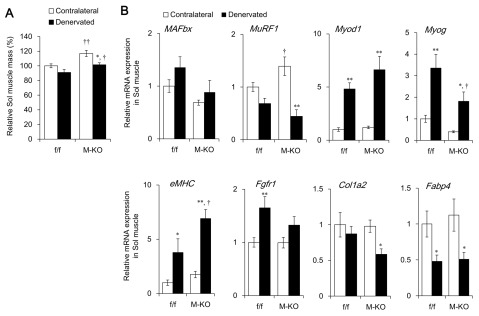
Extent of soleus (Sol) muscle atrophy induced by denervation in muscle-specific *Bmal1* knockout (M-KO) mice and littermate control (f/f) mice. **(A)** Relative Sol muscle mass of 7 – 8-month-old (adult) f/f and M-KO mice at seven days after denervation. Sciatic nerves in left hind legs were dissected and contralateral legs served as a control. Value of contralateral Sol muscle weight/body weight in f/f mice is shown as 100%. **(B)** Messenger RNA expression of genes associated with muscle atrophy or degeneration in Sol muscle at seven days after denervation. Data are expressed as means ± SEM (f/f, n = 11; M-KO, n = 9). Value for contralateral muscle of f/f mice is expressed as 1.0. *P < 0.05 and **P < 0.01 for contralateral vs. denervated muscle. ^†^*P* < 0.05 and ^††^*P* < 0.01 for f/f vs. M-KO mice. Sol, soleus.

**Figure 4:**
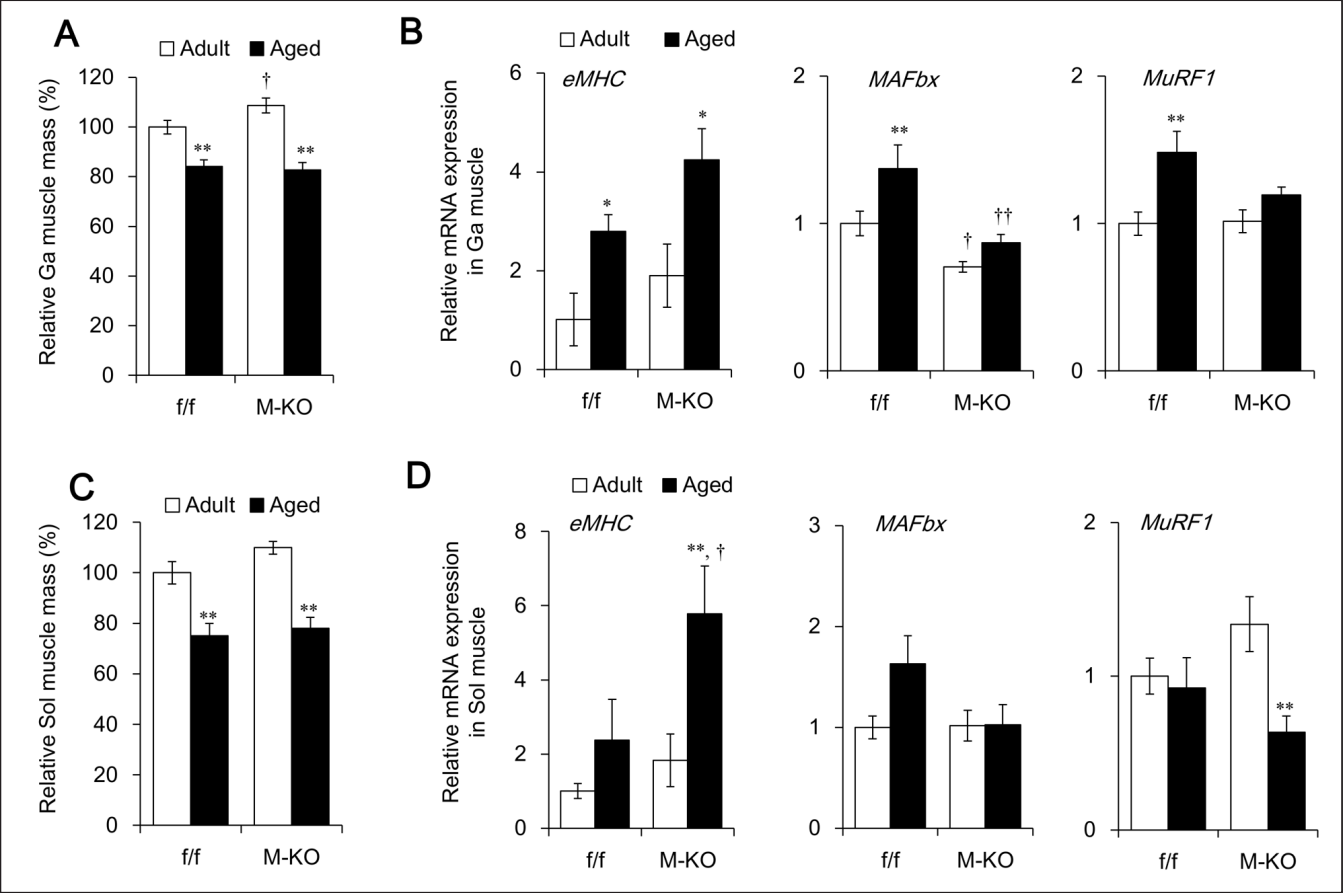
Extent of muscle atrophy induced by aging in muscle-specific *Bmal1* knockout (M-KO) mice and littermate control (f/f) mice. Relative gastrocnemius (Ga) **(A)** and soleus (Sol) **(C)** muscle mass of 7 – 8-month-old (adult) and 23 – 24-month-old (aged) f/f and M-KO mice, respectively. Ratio of muscle weight/body weight of adult f/f mice is shown as 100%. Messenger RNA expression of genes associated with muscle atrophy or degeneration in Ga **(B)** and Sol **(D)** muscles. Data are expressed as means ± SEM (adult f/f, n = 10; adult M-KO, n = 8; aged f/f, n = 6; aged M-KO; n = 6). The value for adult f/f mice is expressed as 1.0. *P < 0.05 and **P < 0.01 for adult vs. aged mice. ^†^*P* < 0.05 and ^††^*P* < 0.01 for f/f vs. M-KO mice. Ga, gastrocnemius; Sol, soleus.

Total RNA was extracted using RNAiso Plus (Takara Bio Inc., Otsu, Japan). Single-stranded cDNA was synthesized using PrimeScript™ RT reagent kits with gDNA Eraser (Takara Bio Inc.). Real-time RT-PCR proceeded using SYBR^®^ Premix Ex Taq™ II (Takara Bio Inc.) and a LightCycler™ (Roche Diagnostics, Mannheim, Germany) with the primer sequences shown in Supplementary Table 1. The amplification conditions were 95°C for 10 s followed by 45 cycles of 95°C for 5 s, 57°C for 10 s and 72°C for 10 s. The amount of target mRNA was normalized relative to that of *Actb*. Values for f/f mice at ZT1, the contralateral muscles of f/f, and adult f/f mice are expressed as 1.0 in Figures 1,2,3 and 4, respectively.

### Statistical analysis

Data were analyzed using Excel-Toukei 2012 software (Social Survey Research Information Co. Ltd., Osaka, Japan) and all values are expressed as means ± standard error of the mean (SEM). Values were assessed using a two-way analysis of variance (ANOVA) followed by Tukey-Kramer multiple comparison tests. Differences were considered significant at *P* < 0.05. We showed the ANOVA results in Supplemental Tables 2–6.

## Results and Discussion

We initially compared the day/night expression of *Bmal1* and other clock genes in the Ga and Sol muscles between f/f and M-KO mice. Rhythmic *Bmal1*mRNA expression was obviously damped in the Ga muscle of M-KO mice (Figure 1A). The rhythmic expression of BMAL1 target genes such as *Per1, Per2, Nr1d1*, and *Dbp* was damped in the Ga muscles (Figure 1A). Levels of *Bmal1* mRNA expression were flattened at the intermediate levels in the Sol muscles of M-KO mice, although the expression of BMAL1 target genes such as *Nr1d1* and *Dbp* was significantly decreased (Figure 1B). The activity of Cre recombinase might be weaker in the Sol (mainly type I fibers), than in the Ga (mainly type II fibers) because we used mice expressing a Cre transgene driven by the Mck promoter, which is preferentially activated in type II fibers [13].

We assessed the effects of denervation-induced muscle atrophy in M-KO mice to determine the functional role of muscle BMAL1. The relative weight of the contralateral Ga muscle to body weight was significantly higher in M-KO, than in f/f mice (Figure 2A). This hypermuscular phenotype might be associated with a decrease in the expression of myostatin, a potent negative regulator of skeletal muscle growth, in M-KO mice (Wada et al., manuscript in preparation). The weight of denervated Ga muscles from f/f and M-KO mice decreased by 18.3% and 17.0%, respectively, compared with the contralateral Ga muscles at seven days after denervation (Figure 2A). The ratio of the loss of muscle mass was essentially identical between f/f and M-KO mice, suggesting that M-KO mice were not resistant and/or susceptible to denervation-induced muscle atrophy. In accordance with this finding, the mRNA expression of *MAFbx* was increased about 2.5-fold in the denervated Ga muscles from both f/f and M-KO mice (Figure 2B). Denervation also significantly induced *MuRF1* mRNA expression in both mouse genotypes, although to a slightly, but significantly attenuated degree in M-KO, compared with f/f mice (Figure 2B). Denervation upregulated the mRNA expression of myogenic differentiation 1 (*Myod1*; a master regulator of myogenesis) 3.5-fold in both f/f and M-KO mice (Figure 2B), although *Myod1* is transcriptionally regulated by BMAL1 [10,14]. Myogenin (Myog) transcription factor transcriptionally regulates *MAFbx* and *MuRF1* gene expression through E-box elements on their promoter regions during denervation-induced muscle atrophy [7]. The expression of *Myog* mRNA was 25-fold upregulated in denervated muscle from f/f, but not M-KO mice (Figure 2B), suggesting that BMAL1 is involved in denervation-induced *Myog* expression independently of its function of facilitating muscle atrophy. The reduced *Myog* expression in denervated M-KO mice might have suppressed the expression of *MuRF1*, but not of *MAFbx*. Transcription factors, including FOXOs, might compensate for decreased *Myog* expression in M-KO mice. The denervation-induced mRNA expression of embryonic myosin heavy chain (*eMHC*) that is associated with muscle regeneration was similar between the mouse genotypes (Figure 2B). Although sustained denervation causes fibrosis in skeletal muscle [15,16], the effect of denervation on the expression of genes associated with fibrosis and fatty degeneration such as fibroblast growth factor receptor 1 (*Fgfr1*), collagen type I alpha 2 (*Col1a2*), and fatty acid binding protein 4 (*Fabp4*) was not significant in either genotype (Figure 2B). We also measured the mass of Sol muscles that mainly comprises slow-twitch muscle fibers. Consistent with the Ga, the contralateral Sol muscle weighed significantly more in M-KO than in f/f mice (Figure 3A). The weight of denervated Sol muscles decreased by 6.9% compared with the contralateral muscles in f/f mice, but the difference did not reach significance. Denervation decreased the weight of the Sol by 12.7% in M-KO mice. These results suggest that muscle *Bmal1* marginally affects the regulation of muscle mass in both fast- and slow-twitch muscles in M-KO mice, although the expression of muscle atrophy- or fibrosis-related genes was partly attenuated (Figure 3). The mRNA expression of *MAFbx* and *MuRF1* was upregulated by denervation in the Ga, but not in the Sol muscle. The extent of *Myog* upregulation also differed between the Ga and Sol muscles. It has been reported that the effects of denervation on the level of these transcripts are greater in the Ga, than in the Sol muscle [17,18]. Susceptibility to denervation-induced atrophy might differ between the Ga and Sol muscles.

Dyar et al. found that muscle histology was normal in M-KO even at the age of 26 months [19], which is in contrast to global *Bmal1* KO mice. We also confirmed that the ablation of muscle *Bmal1* scarcely affected the extent of the aging-induced loss of muscle mass (sarcopenia). The relative weight of the Ga and Sol muscles did not significantly differ between aged (23 – 24 months) f/f and M-KO mice (Figure 4A and C). Furthermore, the expression of *eMHC*, *MAFbx* and *MuRF1* mRNA was also scarcely affected by *Bmal1* ablation (Figure 4B and D).

The present study showed that denervation and aging resulted in a similar progression of muscle atrophy between M-KO and f/f mice, whereas the global deletion of *Bmal1* caused extreme muscle loss in younger mice [9]. Furthermore, untreated (contralateral) Ga and Sol muscles weighed significantly more in M-KO, than in f/f mice, although global KO of *Bmal1* causes severe muscle atrophy [9,10]. We recently described that many genes involved in lipid metabolism and protein degradation are transcriptionally regulated in a circadian manner by systemic neural signals in skeletal muscle [18]. Therefore, the extreme muscle loss in global *Bmal1* KO seemed to be caused by disruption of the central clock in the SCN.

## Supplementary Files

The supplementary files for this article can be found as follows:
