# Supplementary material for: Muscle Bmal1 is Dispensable for the Progress of Neurogenic Muscle Atrophy in Mice
Source: J Circadian Rhythms. 2016 Aug 19;14:6. doi: 10.5334/jcr.141 (PMC5038093; doi:10.5334/jcr.141)
Supplement: Supplementary file 1 [file jcr-14-141-s1.pdf]

Supplemental Table 1. Primer sequences for real-time RT-PCR.

Gene	Direction	Primer sequence (5’ to 3’)
Bmal1	Forward	ATGCAGAACACCAAGGAAGG
	Reverse	CCATCCTTAGCACGGTGAGT
Clock	Forward	ACCACAGCAACAGCAACAAC
	Reverse	GGCTGCTGAACTGAAGGAAG
Per1	Forward	CCAGATTGGTGGAGGTTACTGAGT
	Reverse	GCGAGAGTCTTCTTGGAGCAGTAG
Per2	Forward	CACTCAGGAGTGCATGGAGGAGA
	Reverse	CTGCTCTTGCACCTTGACCAGGT
Nr1d1	Forward	CCCTGGACTCCAATAACAACACA
	Reverse	GCCATTGGAGCTGTCACTGTAG
Dbp	Forward	GGAACTGAAGCCTCAACCAAT
	Reverse	CTCCGGCTCCAGTACTTCTCA
MAFbx	Forward	GGAAGCTTTCAACAGACTGGA
	Reverse	CTCAGGGATGTGAGCTGTGA
MuRF1	Forward	ACGAGAAGAAGAGCGAGCTG
	Reverse	CTTGGCACTTGAGAGGAAGG
Myod1	Forward	TACCCAAGGTGGAGATCCTG
	Reverse	GCATCTGAGTCGCCACTGTA
Myog	Forward	CTACAGGCCTTGCTCAGCTC
	Reverse	ACGATGGACGTAAGGGAGTG
eMHC	Forward	ACTTCACCTCTACCGGATG
	Reverse	ATTGTCAGGAGCCACGAAA
Fgfr1	Forward	CCAGTGCATCCATGAACTCTG GGGTTCTCC

	Reverse	GGTCACACGGTTGGGTTTGTCCTTATCCAG
Col1a2	Forward	CAGAACATCACCTACCACTGCAA
	Reverse	TTCAACATCGTTGGAACCCTG
Fabp4	Forward	CCGCAGACGACAGGA
	Reverse	CTCATGCCCTTTCATAAACT
Actb	Forward	CACACCTTCTACAATGAGCTGC
	Reverse	CATGATCTGGGTCATCTTTTCA

Supplementary Table 2. Results of Two-way ANOVA of clock gene expression profiles.

Muscle	Gene	Genotype	ZT	Genotype × ZT
Gastrocnemius	*Bmal1*	< 0.01	0.023	0.211
	Clock	< 0.01	0.970	0.512
	Per1	0.051	0.054	0.601
	Per2	< 0.01	< 0.01	< 0.01
	Nr1d1	< 0.01	< 0.01	< 0.01
	Dbp	< 0.01	< 0.01	< 0.01
Soleus	*Bmal1*	0.629	< 0.01	< 0.01
	Clock	< 0.01	0.639	0.036
	Per1	0.416	0.092	0.776
	Per2	< 0.01	< 0.01	0.075
	Nr1d1	< 0.01	0.079	0.033
	Dbp	< 0.01	< 0.01	< 0.01

Supplementary Table 3. Results of Two-way ANOVA of muscle weight in denervation experiment.

Muscle	Genotype	Denervation	Genotype × Denervation
Gastrocnemius	< 0.01	< 0.01	0.636
Soleus	< 0.01	< 0.01	0.369

Supplementary Table 4. Results of Two-way ANOVA of gene expression profiles in denervation experiment.

Muscle	Gene	Genotype	Denervation	Genotype × Denervation
Gastrocnemius	*MAFbx*	0.217	< 0.01	0.319
	MuRF1	0.149	< 0.01	< 0.01
	Myod1	0.785	< 0.01	0.342
	Myog	< 0.01	< 0.01	< 0.01
	eMHC	0.240	< 0.01	0.074
	Fgfr1	0.652	0.229	0.759
	Col1a2	0.360	0.968	0.619
	Fabp4	0.433	0.094	0.836
Soleus	*MAFbx*	0.022	0.099	0.632
	MuRF1	0.542	< 0.01	0.016
	Myod1	0.120	< 0.01	0.212

Myog	0.014	< 0.01	0.263
eMHC	0.021	< 0.01	0.147
Fgfr1	0.282	< 0.01	0.298
Col1a2	0.216	0.041	0.284
Fabp4	0.638	< 0.01	0.767

Supplementary Table 5. Results of Two-way ANOVA of muscle weight in aging experiment.

Muscle	Genotype	aging	Genotype × aging
Gastrocnemius	0.135	< 0.01	0.189
Soleus	0.331	< 0.01	0.290

Supplementary Table 6. Results of Two-way ANOVA of gene expression profiles in aging experiment.

Muscle	Gene	Genotype	aging	Genotype × aging
Gastrocnemius	*eMHC*	0.065	< 0.01	0.645
	MAFbx	< 0.01	< 0.01	0.260
	MuRF1	0.148	< 0.01	0.110
Soleus	*eMHC*	0.013	< 0.01	0.117
	MAFbx	0.106	0.080	0.090
	MuRF1	0.876	0.020	0.056
